# End of Life Care for Patients Dying of Stroke: A Comparative Registry Study of Stroke and Cancer

**DOI:** 10.1371/journal.pone.0147694

**Published:** 2016-02-04

**Authors:** Heléne Eriksson, Anna Milberg, Katarina Hjelm, Maria Friedrichsen

**Affiliations:** 1 Department of Social and Welfare Studies, Linköping University, Norrköping, Sweden; 2 Palliative Education & Research Centre, Vrinnevi Hospital, Norrköping, Sweden; 3 Department of Advanced Home Care, Vrinnevi Hospital, Norrköping, Sweden; Osaka University Graduate School of Medicine, JAPAN

## Abstract

**Background:**

Although stroke is a significant public health challenge and the need for palliative care has been emphasized for these patients, there is limited data on end-of-life care for patients dying from stroke.

**Objective:**

To study the end-of-life care during the last week of life for patients who had died of stroke in terms of registered symptom, symptom management, and communication, in comparison with patients who had died of cancer.

**Design:**

This study is a retrospective, comparative registry study.

**Methods:**

A retrospective comparative registry study was performed using data from a Swedish national quality register for end-of-life care based on WHO`s definition of Palliative care. Data from 1626 patients who had died of stroke were compared with data from 1626 patients who had died of cancer. Binary logistic analyses were used to calculate odds ratios, with 95% CI.

**Results:**

Compared to patients who was dying of cancer, the patients who was dying of stroke had a significantly higher prevalence of having death rattles registered, but a significantly lower prevalence of, nausea, confusion, dyspnea, anxiety, and pain. In addition, the stroke group had significantly lower odds ratios for health care staff not to know whether all these six symptoms were present or not. Patients who was dying of stroke had significantly lower odds ratio of having informative communication from a physician about the transition to end-of-life care and of their family members being offered bereavement follow-up.

**Conclusions:**

The results indicate on differences in end-of-life care between patients dying of stroke and those dying from cancer. To improve the end-of-life care in clinical practice and ensure it has consistent quality, irrespective of diagnosis, education and implementation of palliative care principles are necessary.

## Introduction

Stroke is a significant public health challenge, and accounts for 10% of all deaths globally. Although there has been a decline in stroke mortality over the past decades [1], almost 40% of the afflicted die within a year after the incident [2], which makes end of life care and palliative treatment essential [3, 4]. Today, it is unclear what kind of end-of-life care that patients dying from stroke receive. Do they receive care equivalent to that of patients suffering from the “traditional palliative care diagnosis”, cancer? The evidence of palliative care for patients within a stroke context is scarce: recommendations rely on research from the cancer context [5].

Although there are international, specific, and detailed guidelines for the management of stroke, they usually focus on the acute phase and the rehabilitation phase, and only briefly mention palliative care for those patients who will not recover despite medical treatment [6, 7]. The predominant culture supporting stroke care relies on an evidence-base for acute and rehabilitative neurological interventions [8], which sometimes is in contradiction to the philosophy underpinning palliative care even though the need and the benefit of palliative care for dying stroke patients and their families have been emphasized [5, 8].

Outside specialist palliative care clinics, there are contradictions and confusions around the term “palliative care”[8, 9], even though there is a World Health Organization (WHO) definition that explains its content [4]. The confusion might be about phases of care as these can be expressed differently, for example, restorative, palliative and terminal phases [7]. In this study we have focused on the last phase of palliative care, previously called the terminal phase. Palliative care has its own definition according to the WHO [4]. In brief, this definition conveys that palliative care is a team approach, that improves the quality of life of patients and their families facing problems associated with life-threatening illness, such as physical, psychosocial and spiritual problems and needs. This is done through the relief of suffering by early identification, assessment and treatment of symptoms. Palliative care should affirm life and treats dying as a normal process, and should neither hasten nor postpone death. The needs of patients and their families are in focus, and they should be offered a support system to help them during the disease trajectory and with their own bereavement, which also includes bereavement counselling for family members after the patient’s death [4]. The phase of palliative care at the end of life might start with a “break point dialogue” that is a communication between the physician, patient and family member about the transition to palliative care at the end-of-life, where content of the continued care is discussed, based on the patient’s condition, needs and desires [10].

Symptom management is essential in palliative care [4], and patients afflicted with stroke even though they are not at the end of life, might suffer from a range of specific symptoms that have a profound impact on their quality of life, for example, pain, spasticity, depression, and anxiety [11–15]. End-of-life care in patients with stroke and their families have been studied to a limited extent, but there are a few reports on stroke patients`last phase of life for example regarding symptoms [11, 12, 15]. Mazzocato et al. found a high prevalence of certain symptoms among dying stroke patients (n = 42), namely, dyspnea (81%) and pain (69%), mouth dryness (62%), and anxiety (26%)[12]. In addition, Ntlholang et al. identified respiratory secretions as a main palliative care symptom in patients dying of stroke (n = 54)[15]. In a recent review study [13] several symptoms were present in the last two weeks of life. The study included patients suffering from cancer, renal disease, heart failure and obstructive pulmonary disease. The symptoms with the highest prevalence were: dyspnea (56.7%), pain (52.4%), respiratory secretions/death rattle (51.4%), and confusion (50.1%)[13]. Still, there is limited data in a larger population concerning stroke and the complexity of palliative care for example symptom prevalence.

Despite the medical improvement in the treatment of stroke, a significant proportion of patients die in the acute phase, 21% within the first 30 days [2]. Life and death decisions after a severe stroke are therefore common in the acute phase after stroke [16]. According to prediction models, it is possible to make a prognostic estimate [14, 17, 18], however, the methods are surrounded by uncertainty, limitations, and potential for bias [19]. Therefore, treatment decisions are often complex [11, 19] for example concerning the initiation or withdrawal of artificial nutrition, and the use of a mechanical ventilator [20–23]. The decision to give palliative care might be delayed, resulting in prolonged and unnecessary suffering and lack of symptom relief for the afflicted. It may also generate ethical dilemmas among the health care staff in the stroke team [23].

Although it is well known that stroke patients suffer from a range of symptoms and that communication near the end of life with health care staff may ameliorate the situation for patients and their families [24], there are only a few reports of studies on patients dying of stroke that have focused on the prevalence of symptoms, managements and appropriate communication according to the principles of palliative care.

To improve end of life in the stroke context and reduce suffering for the afflicted and their families [25], it is important to determine what kind of end of life care will be given, for example, in terms of what symptoms are prevalent in the last phase of life, how they are managed, and to what extent health care staff communicate about imminent death with dying patients and their families. The availability of a national quality register for end-of-life care, the Swedish Register of Palliative care (SRPC), offers a unique opportunity to perform such a study in a larger population. The purpose of this register is to measure how the actions of different care units correspond to established goals for care at the end of life [26], according to the WHO`s definition of palliative care [4]. In this study we wanted to compare the end-of-life care of patients who died of stroke with patients who died of cancer. The primary, aim of this study was to study end-of-life care during the last week of life for patients dying of stroke, in terms of symptom prevalence, symptom management, and communication with patient and the family, and the secondary aim was to compare the results with those who died of cancer.

## Materials and Methods

### Design

This study is a retrospective, comparative registry study.

### Quality of palliative care assessment

SRPC received the status of a national quality registry in 2006 and has been evaluated, validated, and revised [26, 27]. In 2012, 62% of all deaths in Sweden were recorded in this registry [28]. The online registration is performed postmortem by a nurse or physician who has been responsible for the previous care, and is based on information from the patient’s medical records and/or health care staff experiences of the patient’s situation on one occasion or more during the last week. The responses to the web questionnaire cannot be submitted unless all questions have been answered; in other words, there were no internal missing values in this study. To be included in the registry, the deaths had to be expected.

The registry includes 30 questions, of which 24 were analyzed, concerning the place of death, main cause of death, symptoms, symptom management during the last week of life, and communication with the patient and the family about transition to end of life care (yes, no, don’t know). The questions regarding symptoms were followed with a question on whether the specific symptom was relieved (fully, partly or not at all). There were three questions concerning the presence of decubitus, satisfaction with the care provided by health staff, and whether the patient`s ability to self-determine was retained until the final days. These were answered on an ordinal scale (graded 0–5).

### Study population

Using the SRPC data, the inclusion criteria for this study were: diseased patients who had stroke as the main cause of death; who had died in hospital or in a nursing home; and whose death was expected. Data were collected during November 2011 to October 2012. A statistician matched each stroke patient with a registered patient who had died of cancer in the same period, according to the place of death, sex and age (Table 1). A total of 3252 persons were included in the study. The mean age of the population was 83.5 years, standard deviation (SD) + 8.5 years. Approximately 61% had died in hospital (n = 995) and 39% (n = 631) had died in a nursing home, and 60% of the population were females.

**Table 1:** Demographic data of the study population (N = 3252), patients who had died of stroke (n = 1626) or cancer (n = 1626) mean age 83.5 years (SD + 8.5 Years).

	Stroke n (%)	Cancer n (%)	N
**Age at time of death**			
<69	104 (7)	104 (7)	208
70–79	279 (17)	279 (17)	558
>80	1243 (76)	1243 (76)	2486
**Sex**			
Female	972 (60)	972 (60)	1944
Male	654 (40)	654 (40)	1308
**Settings of death**			
Nursing home	631 (39)	631 (39)	1262
Hospital	995 (61)	995 (61)	1990

### Ethics statement

This study was carried out in accordance with the principles of the Helsinki Declaration [29] and the research protocol was approved by the Regional Ethical Review Board at Linkoping University, Sweden (Permit Number 2012/250-31). All patients were assessed by physicians or nurses in charge, after their death, and were then reported to SRPC. All patients in SRPC are de-identified, no name and other personal identification can be found.

### Statistical analysis

Descriptive statistics were calculated for the individual variables with means and SD for continuous variables, and frequencies and percentages for categorical data. All data were dichotomized and binary logistic regression analyses were used to calculate odds ratios (ORs) with 95% confidence intervals (CIs) in univariate models. A p-value of <0.05 was considered to be statistically significant. IBM SPSS Statistics version 23 was used to perform the statistical analysis.

## Results

### Prevalence of symptoms and symptom management

All six assessed symptoms were prevalent in both studied groups (on one occasion or more during the last week). Compared to the patients with cancer, the patients dying of stroke had significantly higher OR for having death rattles (OR 1.7;95% CI 1.47–1.96), but lower ORs for registering the symptoms of pain, nausea, confusion, anxiety and dyspnea (Tables 2–3). However, the stroke group had significantly higher ORs for health care staff not to know, whether all of the symptoms in the questionnaire were present or not: pain (OR 8.84;95% CI 6.25–12.50) confusion (OR 2.59;95% CI 2.16–3.09), anxiety (OR 2.41;95% CI 2.16–3.09) (Tables 2–3). The stroke group had a significantly higher OR for having individual on—demands prescription for relief of death rattles (OR 1.25;95% CI 1.04–1.50) but significantly lower ORs for having individual on—demands prescription for relief of; pain (OR 0.37;95% CI 0.28–0.48), nausea (OR 0.37;95% CI 0.32–0.43) anxiety (OR 0.57;95% CI 0.48–0.67) (Tables 2–3). In addition, the stroke group had significantly higher ORs for healthcare staff not knowing whether such on-demand prescriptions were present or not, where pain had the highest OR (OR 2.80;95% CI 1.30–6.02). The OR were higher for having nutrition supply in the stroke group on the last day of life (OR 1.35;95% CI 1.14–1.59), compared with the cancer patients.

**Table 2:** Comparison of registered symptoms and symptom management during the last week of life in patients who died of stroke and patients who died of cancer.

Variables	Stroke n (%)	Cancer n (%)	*P*-value	Odds Ratio
	n = 1626	n = 1626		(95% CI)
**Presence of dyspnea**	265 (16.3)	379 (23.3)	<0.001	0.69 (0.58–0.82)
No	1167 (71.8)	1158 (71.2)		
Do not know	194 (11.9)	89 (5.5)	<0.001	2.34 (1.80–3.03)
Fully relieved	74 (27.9)	126 (33.2)	0.151	0.77 (0.55–1.09)
**Presence of death rattles**	987 (60.7)	800 (49.2)	<0.001	1.70 (1.47–1.96
No	574 (35.3)	792 (48.7)		
Do not know	65 (4.0)	34 (2.1)	<0.002	1.95 (1.28–2.96)
Fully relieved	417 (42.2)	341 (42.6)	0.873	0.98 (0.81–1.18)
Prescribed drug injection on demand	1345 (82.7)	1305 (80.3)	<0.017	1.25 (1.04–1.50)
No	247 (15.2)	300 (18.5)		
Do not know	34 (2.1)	21 (1.3)	0.080	1.63 (0.94–2.82)
**Presence of pain**	695 (42.7)	1268 (78.0)	<0.001	0.27 (0.23–0.31)
No	647 (39.8)	320 (19.7)		
Do not know	284 (17.5)	38 (2.3)	<0.001	8.84 (6.25–2.50)
Presence of severe pain, VAS >6 (in the whole group = 1626)	74 (4.6)	371 (22.8)	<0.001	0.15 (0.12–0.20)
Fully relived	556 (80.0)	894 (70.5)	<0.001	1.67 (1.34–2.08)
Prescribed drug injection on demand	1400 (86.1)	1535 (94.4)	<0.001	0.37 (0.28–0.48)
No	201 (12.4)	82 (5.0)		
Do not know	25 (1.5)	9 (0.5)	<0.008	2.80 (1.30–6.02)

**Table 3:** Comparison of registered symptoms and symptom management during the last week of life in patients who died of stroke and patients who died of cancer.

Variables	Stroke n (%)	Cancer n (%)	P-value	Odds Ratio
	n = 1626	n = 1626		(95%CI)
**Presence of anxiety**	308 (18.9)	687 (42.3)	<0.001	0.37 (0.31–0.43)
No	866 (53.3)	715 (44)		
Do not know	452 (27.8)	224 (13.8)	<0.001	2.41 (2.01–2.87)
Fully relieved	225 (73.1)	396 (57.6)	<0.001	1.99 (1.48–2.67)
Prescribed drug injection on demand	1098 (67.5)	1277 (78.5)	<0.001	0.57 (0.48–0.67)
No	476 (29.3)	317 (19.5)		
Do not know	52 (3.2)	32 (2.0)	0.029	1.65 (1.0–2.6)
**Presence of confusion**	129 (7.9)	383 (23.6)	<0.001	0.33 (0.27–0.41)
No	1028 (63.2)	1023 (62.9)		
Do not know	469 (28.8)	220 (13.5)	<0.001	2.59 (2.16–3.09)
Fully relieved	28 (21.7)	62 (16.1)	0.152	1.44 (0.87–2.37)
**Presence of decubitus**	235 (14.5)	232 (13.8)	0.609	1.19(0.60–2.33)
No	17 (1.0)	20 (1.2)		
Do not know	1374 (84.5)	1374 (84.5)	0.934	0.99 (0.81–1.20)
**Assessment of status of mouth was performed**	1059 (65.1)	1014 (62.4)	0.033	1.21(1.01–1.44)
No	309 (19)	358 (22)		
Do not know	258 (15.9)	254 (15.6)	0.847	1.01(0.84–1.23)
**Examination by physician during last days of life.**	1317 (81)	1292 (79.5)	0.175	1.13(0.94–1.36)
Do not know	44 (2.7)	39 (2.4)	0.578	1.13 (0.73–1.75)
**Team were satisfied with the end-of life care**	1481 (91.1)	1462 (89.9)	0.256	1.14 (0.90–1.44)
**Nutrition supply during last day of life**	402 (24.7)	322 (19.8)	<0.001	1.35 (1.14–1.59)
No	1188 (73.1)	1287 (79.2)		
Do not know	36 (2.2)	17 (1.0)	0.010	2.14 (0.19–3.83)

### Communication with patients and their families

Compared to the cancer group the stroke group had a significantly lower OR of having informative communication from a physician about the transition to end of life, (OR 0.09;95% CI 0.08–0.11) (Table 4). The health care staff of the stroke group more often did not know if the place of death conformed to the latest wish of the patient, (OR 2.16;95% CI 1.86–2.51). In addition, the OR for the health care staff not to know if there was a “person present at the time of death” was significantly higher for the stroke group (OR 2.60;95% CI 1.54–4.38). Compared with the family members in the cancer group, the family members in the stroke group had a significantly lower OR of being offered bereavement follow-up contact, (OR 0.67;95% CI 0.57–0.80). In addition, the OR was higher (OR 1.29;95% CI 1.12–1.50) for the healthcare staff not to know if such contact had been offered for the stroke groups`families compared to the cancer groups families.

**Table 4:** Comparison of communication with patients dying of stroke, and their families, and patients dying of cancer, and their families.

Variables	Stroke n (%)	Cancer n (%)	*P*-value	Odds Ratio
	n = 1626	n = 1626		(CI 95%)
**Ability to self-determine retained until the last days**				
Yes	1192 (73.3)	1209 (74.3)	0.453	0.93 (0.78–1.11)
No	335 (20.6)	318 (19.5)		
Do not know	99 (6.1)	99 (6.1)	1.000	1.00 (0.75–1.33)
**Person present with the patient at time of death**				
Yes	1273 (78.2)	1326 (81.5)	0.205	0.89 (0.74–1.06)
No	302 (18.6)	280 (17.2)		
Do not know	51 (3.1)	20 (1.2)	<0.001	2.60 (1.54–4.38)
**Bereavement follow-up was offered to the family**				
Yes	504(30.9)	659 (40.5)	<0.001	0.67 (0.57–0.80)
No	512(31.4)	453 (27.8)		
Do not know	610(37.5)	514 (31.6)	<0.001	1.29 (1.12–1.50)
**Informative communication about transition to end of life care with the patient was given**				
Yes	236 (14.5)	849 (52.2)	<0.001	0.09 (0.08–0.11)
No	1127(69.3)	391 (24.0)		
Do not know	263 (16.2)	386 (23.7)	<0.001	0.62 (0.52–0.73)
**Informative communication about transition to end of life care with family was given**				
Yes	1195 (73.5)	1170 (72)	0.005	0.75 (0.62–0.92)
No	281 (17.3)	208 (12.8)		
Do not know	131 (8.1)	229 (14.1)	<0.001	0.53 (0.42–0.67)
Did not have family	19 (1.2)	19 (1.2)		
**Place of death conformed to the last wishes of the patient**				
Yes	332 (20.4)	538 (33.1)	0.231	1.21 (0.88–1.67)
No	69 (4.2)	136 (8.4)		
Do not know	1225 (75.3)	952 (58.8)	<0.001	2.16 (1.86–2.51)

## Discussion

This study is unique as it identified what kind of palliative care at the end of life patients dying of stroke receive in comparison to patients dying of cancer.

All six studied symptoms were reported as prevalent in the stroke group, from nausea at 7.6% to death rattles at 60.7%. One of the assessed symptoms was pain, which approximately 43% of the stroke patients suffered from during their last week of life, and 5% were registered having severe pain (NRS > 6 on one occasion or more during the last week). Mazzocato et al.`study of patients cared for by an organized palliative care consulting team showed an even higher prevalence of pain in dying stroke patients, namely, 69%[12].

Although the stroke group had a lower registered prevalence for most of the studied symptoms compared with the cancer group, the dying stroke patients suffered more often from death rattles during the last week of life. However, there were no significant difference if death rattles was fully relieved between the groups. Death rattles can be very distressing for family members to listen to, and successful palliation of this symptom has been shown to provide a positive experience for the family [30]. There is still room for improvement regarding this symptom.

Another noteworthy result is that, the stroke group had a significantly higher odds ratio than the cancer group for the staff members not to know whether the patient was suffering from all the studied symptoms or not, with ORs ranging from 1.95 for death rattles to 8.84 for pain. Symptom management is essential in palliative care [4], and before the staff members can take further action to relieve a symptom, it has to be identified and assessed.

In the statement from the American Stroke Association, the understanding of the considerable unmet needs of symptom management for patients dying from stroke is obvious [22]. The use of a specific symptom assessment checklist developed for patients with cognitive impairment who are unable to communicate may be of help in clinical practice to treat needs in end- of- life stroke care, for example by using Doloplus-2 and other visual pain instruments [31, 32] or by using the Palliative Care Needs Checklist [33].

The results of this study also indicate that stroke patients received nutrition supply significantly more often than the cancer patients on the last day of life. This could constitute adequate care, but could also be an expression of decision-making problems concerning how to diagnose impending death and provide optimal end of life care [12, 23]. Today, there are several criteria that clinicians can apply to determine which patients that have a higher risk of dying of stroke, such as severe dysphagia, old age, male sex, hemorrhagic stroke, high C-reactive protein on admittance and a high score on the National Institutes of Health Stroke Scale (NIHSS)[14, 17, 18]. These prognosticators might support both nurses and physicians in clinical decision—making and resource allocation. Still it is important to evaluate each individual patient within his/her typical context.

Another essential part of palliative care is communication with patients and families and within the team [4]. In the current study, the communication with the patient differed between the two groups; 69% of the stroke group did not receive communication about transition to end of life, compared with 24% in the cancer group. A possible explanation for these differences is the degree of uncertainty involved in treatment decisions after severe stroke. In most severe strokes, decisions are made when the prognosis is uncertain and when the outcome is unknown [19], and such uncertainty may inhibit the physician from initiating communication related to a possible transition to end of life care. Also dying stroke patients’ level of consciousness may contribute, but unexpectedly in the current study, there was no significant difference in the results between the studied groups`ability to self-determine the final days. As stroke patients were able to self-determine, it is noteworthy that the health care staff did not know about their symptoms.

The results of the current study also relate to another significant aspect of palliative care [4] namely, the health care staff members support for the patient`s family. In the present study, the families of patients who had died of stroke, had a significantly lower prevalence of being offered bereavement follow-up contact than the families of the cancer group (30.9% vs. 40.5%). A previous study [30] showed that families`experience of stroke, which may strike suddenly and unexpectedly, is traumatic and leads to assimilation difficulties regarding information. Even if the prognosis is uncertain, Payne et al. found that patients and family members required honest and clear information, and wished to be included in an ongoing dialogue [34]. Therefore communication is important, both during ongoing care and afterwards.

The results of the present study raise questions if there is a need of improvement in stroke care the last week in life. We cannot be sure, as our results does not necessarily present the reality, but the results indicate differences in assessment for all studied symptoms. Does this mean that there is a need of a development and implementation of palliative care skills within the health care structure that has previously been suggested by other authors [35]? Settings with a combination of curative/restorative intention need to go hand in hand with the culture of palliative care [33], to enhance an ethical thinking [3] and improve the quality of end of life care.

Further studies are needed concerning whether an educational intervention would influence end of life care for stroke team members and affect patients’ end of life care. However, family members’ perspectives on the care provided during the last week of life are also important.

### Strength and limitations

A few issues ought to be raised in terms of the limitations of this study. First of all, there were six symptoms registered in the questionnaire; other possible symptoms that might occur were not reported in this study. Secondly, it seems worth noting that the questionnaires were completed retrospectively, so recall bias could have affected the results. There might be selection bias in the units that are registered in the SPRC, namely, units particularly interested in palliative care. In addition, to avoid confounders regarding age, sex and caregivers the groups were matched. Finally, the validity of the questionnaire has been continually evaluated [26, 27].

## Conclusions

This study has identified differences in palliative care at the end of life between patients dying of stroke compared with patients dying of cancer especially regarding symptoms, symptom management, communication with the patient, and offering a bereavement follow-up to the family. The results may have implications for clinical practice. Does health care staff need to pay more attention to the palliative care needs of patients dying of stroke and to their family members? Further studies in this area are needed to determine this.
